# Effector Caspases and Leukemia

**DOI:** 10.1155/2011/738301

**Published:** 2011-04-14

**Authors:** Ying Lu, Guo-Qiang Chen

**Affiliations:** Department of Pathophysiology, Key Laboratory of Cell Differentiation and Apoptosis of Ministry of Education, Shanghai Jiao Tong University School of Medicine, Shanghai 200025, China

## Abstract

Caspases, a family of aspartate-specific cysteine proteases, play a major role in apoptosis and a variety of physiological and pathological processes. Fourteen mammalian caspases have been identified and can be divided into two groups: inflammatory caspases and apoptotic caspases. Based on the structure and function, the apoptotic caspases are further grouped into initiator/apical caspases (caspase-2, -8, -9, and -10) and effector/executioner caspases (caspase-3, -6, and -7). In this paper, we discuss what we have learned about the role of individual effector caspase in mediating both apoptotic and nonapoptotic events, with special emphasis on leukemia-specific oncoproteins in relation to effector caspases.

## 1. Introduction

The original investigations showed that CED-3 and CED-4 genes play essential roles in either the initiation or execution of the cell death program during the development of the model organism nematode Caenorhabditis* elegans* (C. elegans). Further study proposed that CED-3 acts as a cysteine protease in controlling the onset of programmed cell death in C. elegans, and the CED-3 protein in C. elegans is similar to human interleukin-1*β* (IL-1*β*) converting enzyme (ICE) gene, a cysteine protease that can cleave the 31 kD inactive precursor of IL-1*β* to generate the active form of cytokine. Overexpression of ICE (currently named caspase-1) is sufficient to induce programmed cell death of mammalian cells, suggesting that members of the CED-3/ICE gene family might function in programmed cell death in vertebrates [1]. With this encouragement, so far fourteen members of mammalian caspase family have been identified [2]. In general, caspase presents within the cell as inactive zymogen that consists of an N-terminal prodomain of variable length, a large subunit (p20), a short linker motif, and a small subunit (p10). In response to apoptotic stimuli, the zymogens are activated through proteolytic processing at specific asparagine residues located within the prodomain, resulting in the generation of active caspases in the form of (p20)_2_–(p10)_2_ heterotetramer. Active caspases subsequently initiate apoptosis or inflammatory responses by the cleavage of specific substrates [2, 3]. Based on the structure and function, the caspase family can be divided into three categories. The caspases bearing larger prodomains are inflammatory caspases (caspase-1, -4, -5, -11, -12, -13, and -14) and initiator/apical/upstream of apoptosis caspases (caspase-2, -8, -9, and -10), while caspases with shorter prodomains are effector/executioner/downstream caspases (caspase-3, -6, and -7). The effector caspases perform the actual destruction of the cell and are proteolytically activated by the apical caspases that initiate the caspase cascade. Caspase zymogen can be activated by the so-called extrinsic pathway through the death receptor signaling complex for procaspases-8 and -10, and the intrinsic mitochondria-mediated pathway through the apoptosome as activating complex for procaspase-9. In addition, caspases can also be activated by the granzyme B-mediated and endoplasmic reticulum (ER) stress-mediated pathways [2–4]. 

The executioner caspases and initiator caspases possess distinct mechanisms of activation-cleavage for the executioners and cleavage-independent dimerization for the initiators [5]. The executioner caspases exist constitutively within the cell as inactive dimers and require cleavage within their interdomain linker for activation. In contrast, the zymogens of the initiator caspases exist as inactive monomers and require dimerization to get activated. Of note, this activation is independent of cleavage, which is different from the common mechanism shared by the executioner caspases and most other protease zymogens. Upon activation, the effector caspases carry out the death signal through cleavage of their specific substrates that leads to activation of other destructive enzymes such as DNases or degradation of structural and signalling proteins within the cell. As specific cysteine proteases, caspases typically recognize XXXD, a four-amino-acid motif with an asparagine as the C-terminal residue and cleaving site. Thus far, more than 390 caspase substrates have been identified, including a number of leukemia-specific proteins that will be discussed later [5–8]. 

## 2. Caspase-3

The human caspase-3 gene, a homology to C. elegans CED-3 gene, encodes a 32 kDa protein and was first cloned from human Jurkat T-lymphocytes in 1994 [9]. Extensive studies have identified caspase-3 as the primary effector caspase in most mammalian cells including leukemia cells [10]. Recently, caspase-3 has been demonstrated to play an important role in determining the cellular sensitivity to diverse apoptotic stimuli, including doxorubicin [11], etoposide [11], and cisplatin [12]. In addition, caspase-3 is involved in a number of nonapoptotic events including proliferation of forebrain cells, keratinocytes and B-cells, MHC II expression and dendritic cell maturation, differentiation of neural stem cells, myoblasts, osteoblasts, platelets, erythroblasts, and lens epithelial cells [13–22]. Caspase-3-dificient mice exhibit strain-specific phenotypes. For example, caspase-3-deficient 129X1/SvJ mice are prenatal lethal and exhibit significant neural precursor cell expansion and exencephaly. In contrast, caspase-3-deficient C57BL/6 mice are viable, fertile and display no apparent brain pathology [23, 24]. 

## 3. Caspase-7

Caspase-7 was independently cloned as ICE-LAP3, Mch3, and CMH-1 in three different laboratories. Due to the high similarity in structure (58%) and substrates specificity with caspase-3, caspase-7 has been considered functionally redundant with caspase-3 [25]. Indeed, similar to caspase-3, caspase-7-deficient mice on a C57BL/6 background have a normal lifespan and display little discernable apoptotic phenotype. However, mice deficient for both caspase-3 and -7 in the same strain die shortly after birth, supporting the functional redundancy of these two caspases during embryogenesis [24, 26]. More intriguingly, several studies using cell-free extracts system or caspase-7-deficient mice revealed that caspase-7 also performs distinct, specialized roles in typical apoptosis, ER stress pathway, and inflammatory responses [25, 27–29]. For instance, Walsh et al. screened 20 different purified substrates and found that 12 of them including Bid, X-linked IAP (XIAP), gelsolin, and caspase-6 were preferentially cleaved by caspase-3, whereas one (cochaperone p23) was more susceptible to proteolytic processing by caspase-7 [27]. 

## 4. Caspase-6

Caspase-6 is not as broadly studied as caspases-3 and -7, but is considered as an effector caspase based on its short prodomain and interdomain cleavage activation mechanism [5]. Caspase-6 substrates include a wide range of proteins involved in cell cycle, survival, or development such as SATB1, p27Kip1, Notch1, AP-2*α*, lamin A, Akt, and 5-lipoxygenase [30–37]. Caspase-6-deficient mice develop normally and are only mildly resisted to anti-Fas-induced apoptosis [38]. Interestingly, caspase-6 has been demonstrated to play critical roles in nonapoptotic procedures including B-cell activation and differentiation, axonal degeneration, and human gastric and colorectal carcinomas development [37, 39, 40]. 

## 5. Leukemia-Associated Fusion Proteins as Substrates of Effector Caspases

Since Nowell and Hungerford discovered Philadelphia chromosome originated from *t*(9;22) in chronic myeloid leukemia (CML) in 1960, chromosomal aberrations have attracted much attention in the field of cancer cytogenetics, particularly in hematologic malignancies [41, 42]. To date, more than 500 recurrent chromosomal aberrations have been identified in leukemia, a frequency much higher as compared to solid tumors [43]. Chromosomal translocations are the most commonly happened and well characterized among all the chromosome disturbances. For instance, the RUNX1 gene, located in chromosome 21q22 and a pivotal regulator of definitive hematopoiesis, has been identified in 17 translocations at the molecular level [44]. The occurrence of subtype-specific chromosomal translocations in leukemia strongly suggests that these aberrations play important roles in the process of carcinogenesis. Moreover, chromosomal translocations are also used as diagnostic and therapeutic markers for leukemia [45, 46]. Based on the important role of these fusion proteins in the initiation and development of specific leukemia, drug-triggered fusion protein inhibition or degradation has been proven to be a successful strategy in the treatment of leukemia. Of great interests, other than ubiquitination, several prevalent fusion proteins have been demonstrated to be degraded as caspase-3 substrates, including PML-RAR*α*, AML1-ETO, BCR-ABL, and TAF15-CIZ/NMP4 [8, 47–49]. More importantly, rather than merely a by-stander apoptotic effect, the proteolysis of these fusion proteins by caspase is mostly involved in nonapoptotic events such as cell differentiation and proliferation. This provides a new mechanism and potential that might help to develop novel strategy to cure fusion protein-associated leukemia. 

### 5.1. PML-RAR*α*

The chromosome translocation *t*(15;17)(q22;q21) is seen in 98% acute promyelocytic leukemia (APL) patients, characterized by a terminal differentiation block of myeloid cell development [45, 50, 51]. This translocation juxtaposes the promyelocytic leukemia (PML) gene on chromosome 15 with the retinoic acid receptor*α* (RAR*α*) gene on chromosome 17 and results in the expression of the PML-RAR*α* fusion protein in hematopoietic myeloid cells. This frequently occurred fusion protein initiates APL by acting as a transcriptional repressor that interferes with genes involved in cell differentiation, apoptosis, and self-renewal [50, 52, 53]. Two therapeutic agents in clinical use for APL, all-transretinoic acid (ATRA), and arsenic trioxide (As_2_O_3_) can target PML-RAR*α* and induce PML-RAR*α* degradation through ubiquitin-proteasome pathway, resulting in a complete remission with the differentiation and apoptosis of leukemic cells [54–56]. Earlier, Nervi et al. found that PML-RAR*α* is cleaved by a caspase-3-like activity induced by ATRA treatment and identified aspartate 522 within the *α*-helix region of the PML component as a caspase-3 cleavage site. Interestingly, inhibition of caspase activity could prevent retinoic acid- (RA-) induced PML-RAR*α* degradation without impairing RA-induced differentiation, suggesting that APL cells may undergo differentiation in the presence of PML-RAR*α* expression [49]. These findings are in agreement with recent data indicating that other than degrade PML-RAR*α*, ATRA is able to induce a switch in PML-RAR*α* activity from a repressor to an activator of myeloid differentiation, possibly by triggering its transcriptional activator function on specific RA-target genes [57–59]. As proposed by the authors, another potential contribution of the degradation of PML-RAR*α* by caspase-3 is to leave the RAR*α* component intact and mediate RA-dependent transcription since the major caspase cleavage site locates within the PML component of the fusion protein [49]. Taken together, the role of cleavage of PML-RAR*α* by caspase-3 might play a critical role in ATRA-induced APL differentiation. 

### 5.2. AML1-ETO

The chromosome translocation *t*(8;21)(q22;q22) was firstly identified by Dr. Janet Rowley in 1973 during the analysis of a leukemia patient sample. It is one of the most frequently occurred genetic abnormalities in acute myeloid leukemia (AML), identified in 15–20% of AML patients and over 40% cases of AML-M2 subtype and rare cases of M0, M1, and M4 subtypes of the French-American-British classification [60, 61]. This translocation leads to expression of the AML1-ETO fusion transcription factor which is composed of the first 177 amino acids of AML1/RUNX1 and almost the entire ETO (also known as RUNX1T1 or MTG8) protein. This fusion protein displays dichotomous function since it not only blocks differentiation but also induces growth arrest and apoptotic susceptibility of leukemic cells [62–64]. Similar to PML-RAR*α*, both apoptosis-independent and -dependent degradation of AML1-ETO have been reported [48, 56, 65–68], but the precise mechanism and biological significance of AML1-ETO degradation remain obscure. Recently, we found that AML1-ETO endows leukemic cells with susceptibility to both extrinsic and intrinsic apoptosis and provided direct evidence showing AML1-ETO as a caspase-3 substrate. Site-directed mutagenesis analyses mapped two aspartates (TMPD^188^ and LLLD^368^) within ETO component as caspase-3-targeted sites in the AML1-ETO sequence. More intriguingly, proteolytic cleavage of AML1-ETO is essential for the apoptosis-enhancing effect of AML1-ETO protein because double mutation of aspartates at 188 and 368 abrogated the apoptosis-amplified action of AML1-ETO completely, while expression of the caspase-3-cleaved AML1-ETO C-terminal fragment is sufficient to enhance apoptotic sensitivity [48].

The mechanism by which AML1-ETO contributes to AML development is not clearly established. Sole expression of AML1-ETO failed to generate leukemia in various murine transgenic models, suggesting that additional genetic events might be necessary for AML1-ETO-positive cells to adopt leukemogenic behavior [69]. Because the effect of full-length AML1-ETO on apoptosis does not favor leukemogenesis, we proposed that mutation of caspase-3-targeted sites that would result in the abrogation of apoptosis-enhancing effect might exist in *t*(8;21)-positive AML patients. In fact, a previously unknown spliced variant transcript of AML1-ETO (AML1-ETO9a) in AML patients encoding a C-terminally truncated AML1-ETO protein, which could induce rapid development of leukemia in murine retroviral transduction-transplantation model, has been identified in a number of AML patients [70]. 

### 5.3. BCR-ABL

The Philadelphia chromosome, resulting from a reciprocal translocation between chromosomes 9q34 and 22q11, generates a 190- or 210-kDa fusion protein BCR-ABL identified in more than 95% of chronic myeloid leukemia (CML) and half of patients with adult-onset acute lymphoblastic leukemia (ALL) [41, 71–73]. The BCR-ABL oncoprotein is a constitutively active tyrosine kinase that endows the leukemic cells with growth advantage. In addition, leukemic blasts expressing BCR-ABL display resistance to apoptosis, lack of cell adhesion, and arrested differentiation [73–76]. Similar to PML-RAR*α*, downregulation of BCR-ABL has been observed during differentiation of leukemic cells, but the mechanism is still largely unknown [77, 78]. Recently, using a K562 cell line transfected with a temperature-sensitive mutant of p53, Cotter group demonstrated that p53-induced erythroid differentiation in K562 cells required caspase activity, which resulted in the cleavage of C-ABL and BCR-ABL tyrosine kinases in the absence of apoptosis [47]. *In vitro* experiment showed that C-ABL and BCR-ABL proteins are targets for caspase-3 and -7 but not for caspase-6 and -8. Interestingly, although C-ABL and BCR-ABL proteins can be detected in both nucleus and cytoplasm/cytoskeleton, the nuclear pool of BCR-ABL and C-ABL proteins is preferentially cleaved by caspase(s) following p53 expression in K562 cells, suggesting that only proteins present in a particular location are targeted for degradation [47].

The central role of BCR-ABL kinase in leukemogenesis promotes it as an ideal target for drug screens to treat CML with the attempts to decrease the amount of the BCR-ABL transcripts and/or to inhibit its tyrosine kinase activity. Imatinib mesylate (Gleevec, STI-571), which blocks the binding of ATP to the activated tyrosine kinase and allows the leukemic cells to differentiate and undergo apoptosis, has revolutionized the treatment of CML [79]. However, development of resistance towards imatinib has been a major limitation particularly in the treatment of advanced-stage CML [46]. Mechanisms underlying drug resistance include overexpression of BCR-ABL and amplification of the BCR-ABL gene, acquired additional genomic alterations, and point mutations within the ABL kinase domain that interfere with imatinib binding [80, 81]. More recently, BCR-ABL alternative splicing has been described in a significant number of CML patients and recognized as a common mechanism for drug resistance [82–84]. Ma et al. described several novel mutations that result in BCR-ABL truncations of various lengths within the kinase domain, leading to mutants missing the ABL C-terminal nuclear localization signal (NLS), DNA- and actin-binding (DB and AB) domains, respectively [84]. How did these truncations occur and their contributions to the drug resistance deserve further investigation. Of note, cleavage at the sequence DTAD, one of the caspase-3-targeted sites within BCR-ABL sequence, would release a 52-kDa fragment, leaving the kinase domain intact [47]. These newly identified kinase domain truncations may provide a novel mechanism associated with drug resistance that is caused by executioner caspase activity. 

### 5.4. TAF15-CIZ/NMP4

TAF15 belongs to a DNA- and RNA-binding protein family, known as the FET (also named TET) family that comprises TLS/FUS (translocated in liposarcoma), EWSR1 (Ewing sarcoma), and TAF15. Members of the FET family are implicated in transcriptional activation, mRNA/microRNA processing, and maintenance of genomic integrity [8, 85, 86]. More intriguingly, the fusion of FET proteins to various transcription factors (i.e., ERG, ATFI, CHOP, FLI-I) has been found in multiple human malignancies including leukemia and solid tumors. Recently, a chromosomal translocation at *t*(12;17)(p13;q11) or its variant *t*(12;22)(p13;q12) resulting in the rearrangement of the EWSR1 or TAF15 with the transcription factor CIZ/NMP4 (Cas-interacting zinc finger protein/nuclear matrix protein 4) was identified in AML [87]. Furthermore, TAF15-CIZ fusion proteins and wild-type TAF15 are demonstrated to be cleaved by caspases-3 and -7 both *ex vivo* and *in vitro. *The cleavage site recognized by these two caspases is ^106^DQPD/Y^110^ [8]. Due to the lack of understanding of TAF15-CIZ fusion protein, the function and significance of its cleavage by caspase-3/7 is largely unknown. It is interesting that v-Src kinase could phosphorylate TAF15 and TAF15-CIZ at a region containing the caspase cleavage site leading to the block of cleavage. Therefore, it is postulated that increased resistance to proteolysis caused by phosphorylation of TAF15-CIZ might be advantageous for cancer cells that lead to leukemogenesis [8, 88]. 

## 6. Effector Caspases as Prognostic Markers in Leukemia Treatment

As the key effector of cellular death, caspase-3 expression/activity has been implicated as a predictor of survival in AML and ALL [89, 90]. However, the results thus far are controversial, and the clinical significance of caspase level in leukemia is still obscure. Using quantitative Western blot analysis, Estrov et al. measured the level of nonactivated caspase-2, -3 and activated (cleaved) caspase-3 in peripheral blood of 185 patients with newly diagnosed AML. They reported that high levels of procaspase-2 and procaspase-3 denoted poor survival, whereas the high level of cleaved caspase-3 correlated with a favorable prognosis, although with merely a marginal significance [89]. In contrast, most other studies yielded negative data in regards to the association between the level of caspase-3 and survival. For example, Campos et al. observed no relationship between procaspase-2 or procaspase-3 and clinical response of AML patients to therapy as assessed by flow cytometry [91]. Svingen et al. also reported level of procaspase-2 or procaspase-3 from bone marrow samples failed to correlate with response of AML patients to chemotherapy [92]. Other than focus on protein level, Liu et al. detected caspase-3 activity in peripheral blood of children with leukemia prior to, and following, the onset of chemotherapy. They found no association with clinical response but a significant correlation between the caspase-3 activity over the first 24 hours following chemotherapy and activities at hours 6 and 24 [93]. 

## 7. Effector Caspases as Therapeutic Targets

A hallmark feature of leukemia and other cancer cells is the ability to escape apoptosis that correlates with chemotherapy resistance. Therefore, there have been enormous efforts in developing new molecules that could reactivate the apoptotic program in tumor cells. Up to now, a number of compounds/peptides/antibodies targeting a diverse range of apoptosis-related molecules are being explored at the preclinical and clinical levels. For instance, small molecules/antisenses/oligonucleotides that target Bcl-2, XIAP, and survivin and antibodies that target death receptors have been approved by US FDA in clinical trials [4, 94]. 

One potential strategy involves the modulation of natural cellular caspase inhibitors such as XIAP, surviving, c-FLIP, and Smac [95–97]. For example, XIAP, a member of IAP (inhibitor of apoptosis) family, is a natural caspase inhibitor that can specifically bind to the active sites of caspase-3 and -7 or the dimer interface of caspase-9. Antisense oligonucleotide of XIAP with a releasing caspase-3 activity effect has entered the phase I clinical trial in cancer treatment [4, 94, 98].

 Another promising strategy is to design or discover small molecules/compounds that directly activate effector caspases. Because the activation of effector caspases, particularly caspase-3, is ultimately involved in most apoptotic events, molecules that directly target the effector caspases would be ideal drug candidates. However, it would be extremely difficult considering their specific physiologic activation mechanism which requires cleavage by apical caspases. Intriguingly, after screening approximately 20,500 compounds, Putt et al. identified PAC-1 (procaspase-activating compound-1), a small molecule that can directly activate procaspase-3 *in vitro* and induce apoptosis of primary colon cancer cells. It can also induce growth arrest of tumors in murine cancer models [99]. Although whether PAC-1 activates caspase-3 through direct mechanism is still of dispute [100, 101], it provides a potential strategy in treating the many cancers including leukemia that express an elevated level of caspase-3. 

## 8. Conclusions

The pivotal role of effector caspases in multiple cancers including leukemia has been extensively investigated and recognized. The close connection of effector caspases with leukemia-associated proteins promotes them as ideal target for treatment. However, due to the dynamic state and specific activation mechanism of caspase, development of compounds of therapeutic value that directly target caspase is far from satisfactory. A better understanding of caspases activation mechanism and newly developed approaches such as chemical biology will help to generate new lead compounds for cancer treatment. Moreover, drug combination including caspases regulators and drugs that target cross talk signaling pathways may be promising and find their way into the clinic. 
